# Editorial: Advances in theranostics of CNS injuries and diseases: from basic research to clinical practice

**DOI:** 10.3389/fnins.2023.1255751

**Published:** 2023-08-15

**Authors:** Bo Li, Jian Hao, Ping Zheng, Guangzhi Ning

**Affiliations:** ^1^Department of Orthopedics, Sun Yat-Sen Memorial Hospital of Sun Yat-Sen University, Guangzhou, China; ^2^Department of Orthopedics, The Second Aliated Hospital, Guangzhou Medical University, Guangzhou, China; ^3^Department of Neurosurgery, Shanghai Pudong New Area People's Hospital, Shanghai, China; ^4^Department of Orthopedics, Tianjin Medical University General Hospital, Tianjin, China

**Keywords:** spinal cord injury-induced immune deficiency syndrome, kyphotic cervical spondylotic myelopathy, traumatic brain injury, acute ischemic stroke, acute cerebral hemorrhage, moyamoya disease

Amid global aging populations, industrialization, and the continued occurrence of diseases and disasters, the incidence of Central Nervous System (CNS) diseases is on the rise. Injuries to the CNS such as stroke, traumatic brain injury, and spinal cord injury are often irreparable, leading to long-term disability. This research theme focuses on the forefront and progress of CNS injury research in these areas, aiming to summarize the studies on the mechanisms of CNS injury and the clinical application of advanced treatments. This editorial seeks to provide an overview of some key research achievements in this field recently, offering a novel perspective on the treatment of CNS injuries.

In the research “*CCR7-mediated T follicular helper cell differentiation is associated with the pathogenesis and immune microenvironment of spinal cord injury-induced immune deficiency syndrome*”, the role of chemokine receptor CCR7 in T follicular helper cells and its relationship to spinal cord injury was probed. The downregulation of CCR7 was found in acute spinal cord injury and was linked to an altered immune microenvironment, characterized by suppressed T cell signaling and activated chemokine pathways. Thus, CCR7 can potentially be used to identify acute spinal cord injury patients and guide treatment decisions (Li et al.).

Highlighting the importance of surgical strategies in treating kyphotic cervical spondylotic myelopathy (KCSM), a study compared the efficacy of anterior decompression with fusion (ADF) vs. posterior decompression with fixation (PDF). In the study, 117 KCSM patients underwent either ADF or PDF, and cervical alignment parameters were analyzed pre and post-surgery. Results indicated that both ADF and PDF provided neurological improvement and significant reduction in axial symptoms, but ADF was more effective in improving cervical alignment. The study underscores the importance of considering each approach's pros and cons when deciding on a surgical plan for KCSM patients (Du et al.).

In a study underscoring the potential of predictive modeling in managing traumatic brain injury (TBI), a genetic algorithm modified back propagation neural network was employed to predict motor function in TBI patients. Using the Fugl-Meyer assessment scale, data from 463 TBI patients was analyzed to create the model. Key findings suggest the model showed a strong correlation of 0.95 between predicted and actual motor function outcomes. This suggests the potential of this model as a tool for risk and prognosis assessment, and aiding clinical decision-making for TBI patients (Dang et al.).

In a study aimed at understanding mortality rates and risk factors in acute ischemic stroke (AIS) patients undergoing endovascular thrombectomy (EVT), analysis of 245 patients found that early mortality occurred in 22.8% of cases. Key determinants of early mortality were recanalization status, NIHSS score 24 h post-EVT, and symptomatic intracerebral hemorrhage, underscoring their importance in mortality prediction. These findings highlight the need for careful monitoring and targeted interventions for these risk factors in AIS patients undergoing EVT (Chen et al.).

In the study “*The impact of blood pressure variability on prognosis and underlying mechanism in acute cerebral hemorrhage*”, researchers evaluated 120 ICH patients post-antihypertensive treatment. They discovered high systolic blood pressure variability within 1 and 24 h linked to poor 90-day mRS scores. The findings also suggested a negative correlation between 24-h SBP variability and cerebral blood flow. This study could guide individualized antihypertensive treatment strategies for acute ICH patients (Sun et al.).

Highlighting the importance of thalamic structure and anastomosis in the progression of moyamoya disease (MMD), a retrospective study was conducted titled “*Thalamic structure and anastomosis in different hemispheres of moyamoya disease*”. The study compared subcortical gray matter volume and angiographic features in symptomatic and asymptomatic hemispheres of MMD patients. Findings demonstrated significant differences in thalamic volume and the incidence of thalamic anastomosis between these hemispheres, suggesting a potential role in predicting stroke risk and understanding disease evolution in MMD patients (Hu et al.).

To understand genetic factors linked to Alzheimer's disease (AD) onset, researchers carried out a study titled “*Identification of candidate genes associated with clinical onset of Alzheimer's disease*”. They utilized microarray data from post-mortem brains of AD patients and healthy controls to identify differentially expressed genes. They discovered 19 potential genes associated with symptomatic AD, four of which (VSNL1, RTN1, FGF12, and ENC1) were identified as critical. These findings could help pinpoint high-risk individuals prone to early AD onset (Liao et al.).

In a systematic review titled “*The prevalence and risk factors of anxiety in multiple sclerosis*”, researchers analyzed studies to determine anxiety prevalence in MS patients and its associated risk factors. They found that around 36% of MS patients suffer from anxiety. Significant risk factors included age, gender, cohabitation, past psychiatric history, depression, lack of MS medication adherence, relapsing-remitting MS, and baseline EDSS score. This could aid in identifying MS patients at a higher risk of anxiety (Zhang et al.).

In the study “*Functional investigation and two-sample Mendelian randomization study of neuropathic pain hub genes obtained by WGCNA analysis*”, researchers leveraged weighted gene co-expression network analysis (WGCNA) and differential expression to uncover neuropathic pain-associated genes. Key findings showed genes IL2, SMELL, CCL4, among others, correlated with cytokine receptor binding, chemokine pathways, and the JAK-STAT cascade. The link between IL2 and neuropathic pain was further validated by a Mendelian randomization study, helping to identify high-risk individuals (Zeng et al.).

The nine investigations included in this Research Topic delve into crucial facets of spinal cord and brain injuries, as well as neurodegenerative diseases. Each study unveils potential diagnostic markers, therapeutic strategies, and understanding disease mechanisms. They explore everything from chemokine receptors in spinal cord injury to predictive modeling in traumatic brain injury, individualized antihypertensive treatment strategies in cerebral hemorrhage, to genetic risk factors in Alzheimer's disease onset. Such comprehensive studies are crucial for devising more effective and personalized treatment plans, and for enhancing our understanding of these debilitating conditions.

## Author contributions

BL: Writing—original draft. JH: Writing—review and editing. PZ: Writing—original draft. GN: Writing—original draft.

